# The relationship between lifestyle, night shift pattern, mental health and the trajectories of nurses’ metabolic diseases: a cohort study of nurses

**DOI:** 10.3389/fpubh.2026.1743516

**Published:** 2026-04-01

**Authors:** Min Yang, Ying Che, Jiaqi Xu, Xueqian Ma, Hongbo Chen, Baohua Li

**Affiliations:** 1 Department of Obstetrics and Gynecology, Peking University Third Hospital, Bejing, China; 2 Health Examination Center of Peking University Third Hospital, Bejing, China; 3 Nursing Department of Peking University Third Hospital, Bejing, China

**Keywords:** growth mixture model, metabolic diseases, nurse, population health, trajectories

## Abstract

**Objective:**

The study aimed to examine the relationship between lifestyle, night shift pattern, mental health and nurses’ metabolic diseases.

**Methods:**

We included 910 nurses from 2018 to 2022. The Growth mixture model was used to identify the trajectories of metabolic diseases among nurses. And multinomial logistic regression was used to examine the relationship between lifestyle, night shift pattern, mental health and the trajectories of metabolic diseases.

**Results:**

Three distinct trajectories were identified: Maintaining-Low, Chronically-High group, Maintaining-Low group. Compared to the Maintaining-low group, the correlates of Chronically-high group were lack of dietary preference for vegetables and exercise. Low depression scores, high anxiety scores, night shift pattern with a slow increase in metabolic diseases.

**Conclusion:**

The changes of the number of metabolic diseases among nurses in China are heterogeneous. Lack of dietary preference for vegetables and exercise are significantly related to nurses’ metabolic disorders. Among nurses with high initial health level, the correlates of the increase in the number of metabolic diseases are not unhealthy lifestyles, but mental health and night shift pattern.

## Highlights

- Understand the relationship between healthy lifestyle, night shift pattren, mental health and progress of nurses’ metabolic diseases.
- Suggest improvements to nurse’s work environment to reduce the risk of metabolic diseases.

## Introduction

The increasing prevalence of metabolic diseases is a global public health problem, which not only causes premature death, but also increases the economic burden (1). “Metabolic diseases” is characterized by a spectrum of metabolic dysregulation processes, including hypertension (HTN), type 2 diabetes mellitus (T2DM), hyperlipidemia (HLD), obesity and non-alcoholic fatty liver disease (NAFLD) (2). In 2019, there were 5 million deaths due to obesity, 4.3 million deaths due to HLD, 1.4 million deaths due to T2DM, 1.1 million deaths due to HTN and 168,969 deaths due to NAFLD (2). There may be a synergistic effect among metabolic diseases. Guembe et al. (3) found that for each additional components of metabolic syndrome, the incidence of major cardiovascular events increased by 22%. Therefore, examining the progress of various metabolic diseases can better reflect the cumulative metabolic risk. With the cumulative effect of chronic diseases being concerned, studies paid attention to measure change in multimorbidity longitudinally and risk factors for multimorbidity trajectory, which could inform future intervention and prevention strategies at critical life course periods and disease progression turning points (4, 5). However, limited studies have examined the changes of the number of metabolic diseases with time and explored the risk factors contribute to metabolic diseases trajectories, especially combination with NAFLD.

Previous studies found risk factors of metabolic diseases include aging, unhealthy diet, sleep disorder, less physical activity, sedentary, smoking, excessive drinking, and psychological problems (6, 7). Nurses are usually considered as a high-risk group of metabolic diseases, because they may have poor sleep (8), unhealthy diet (9), stress, anxiety and depression (10) due to night shift and heavy workload. Many studies mainly focused on the occurrence of nurses’ metabolic diseases at a certain point in time, but little was known about the longitudinal changes of the number of metabolic diseases and the impact of healthy lifestyle, night shift and mental health on long-term changes. Therefore, this study aimed to examine the trajectories of nurses’ metabolic diseases and explore the relationship between healthy lifestyle, night shift pattern and mental health and the trajectories of nurses’ metabolic diseases, so as to identify individuals with high risk of developing metabolic diseases.

## Methods

### Study design and sample

**Figure 1:**
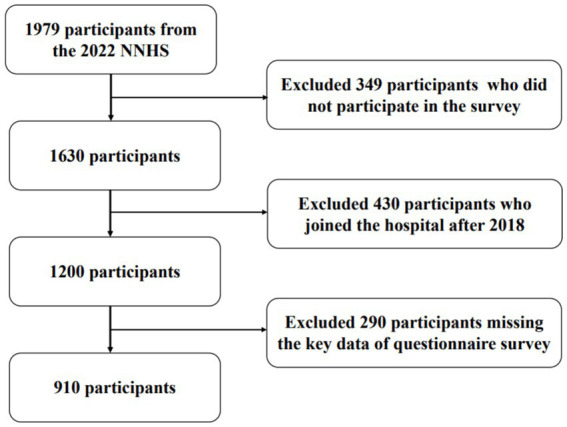
Flow chart of participants.

In this retrospective cohort study, data came from the Chinese Nurse Cohort Study (The National Nurse Health Study, NNHS), which is a WEB-based ambispective cohort study for nurses in China. The cohort included annual physical examination data and questionnaire survey data including sociodemographic, lifestyle, work situation and psychological health, etc. Detailed information for this cohort was included in the protocol for the study (11). The NNHS has been approved by the Medical Research Ethics Committee of Peking University Third Hospital (IRB00006761-M2020306). To ensure the comprehension of the study methodology and results, this study was reported in accordance with the Guidelines for STROBE (12). Due to time constraints, all questionnaire data in this study were collected in 2022. Looking back at the physical examination data from 2022 and before, we found that all the physical examination items before 2018 lacked blood pressure or blood sugar, so we chose the data from 2018 to 2022 for analysis. In 2022, there were 1979 nurses who participated in the physical examination, of which 349 participants were excluded because they did not participate in the questionnaire survey. Some participants (*n* = 430) joined the hospital after 2018. In addition, some participants (*n* = 290) missed the key data in the five-year physical examination. The final analytical sample included 910 nurses (see Figure 1).

### Measures

#### Metabolic diseases

Metabolic diseases included HTN, hyperglycemia, HLD, overweight/obesity, and NAFLD in our study. The disease information is mainly based on the patient’s past medical records, supplemented by physical examination data. There are diagnostic criteria for related diseases based on physical examination data (See Table 1). For each year, possible the number of metabolic diseases ranged from 0 to 5.

**Table 1:** Diagnostic criteria for metabolic diseases.

Metabolic diseases	Diagnostic criteria
Hypertension	≥130/80mmHg (32)
Hyperglycemia	Fasting plasma glucose (FPG) ≥ 6.1 mmol/L (110 mg/dL) (33)
Hyperlipidemia	Total cholesterol (TC) > 5.2mmo1/L; Triglyceride (TG) > 1.7mmo1/L; Low density lipoprotein cholesterol (LDL-C) > 3.4mmo1/L; High density lipoprotein cholesterol (HDL-C) < 1.0mmo1/L (34)
Overweight/obesity	BMI ≥ 24 kg/m^2^ (35)
Non-alcoholic fatty liver disease	(1) no history of drinking alcohol or the alcohol equivalent amount was less than 70 g/week (female) or 140 g/week (male); (2) diseases that can lead to fatty liver such as viral hepatitis, drug-induced liver disease, and autoimmune liver disease were excluded; (3) imaging of diffuse hepatic steatosis (36)

#### Healthy lifestyle, night shift pattern and mental health

Healthy lifestyle included exercise (yes/no), smoking (yes/no), drinking (yes/no), regular diet (yes/no), dietary preference for vegetables (yes/no) and length of sleep (7-8 h/other). Dietary preference for vegetables was defined as a self-reported eating habit among nurses in which both the degree of liking for vegetables and their intake amount are greater than meat and staple food. Night shift pattern included 8-h night shift (nurses are typically required to work back-to-back night and evening shifts), 12-h night shift (5 p.m.-9 a.m., with a 4-h rest break), and extended night shifts (5 p.m.-8 a.m., with no rest break). Mental health included depression and anxiety. The 90-item Hopkins Symptom Checklist (SCL-90) depression and anxiety sub-scales were used as a diagnostic proxy for depression and anxiety in public health surveys, both of sub-scales Cronbach′s alpha were 0.8 (11).

#### Covariate

We controlled for a number of confounding sociodemographic factors, included age, gender, education level, marital status and years of service.

### Statistical analysis

IBM Statistical Version 25.0 (SPSS, Chicago, USA) and R software Version 4.2.1 (R Foundation, Vienna, Austria) containing Packages “lcmm” were used for statistical analysis.

First, we used a growth mixture model (GMM) to identify the trajectories in metabolic diseases. We used the following model fitting indexes and statistical standards to select the best model: (1) Bayesian Information Standard (BIC), akaike’s information criterion (AIC) values and entropy to evaluate the model fitting. Lower values of AIC and BIC, along with higher entropy values, indicated better model fit; (2) The average posterior probability of the group distribution that measures the probability of individual belonging to the specific trajectory group should be greater than 0.7; (3) the number of members in each track group should be greater than 5%. For classified variables, using the chi-square test was used for analyzing, and continuous variables were analyzed by independent sample t tests or ANOVA. Finally, we used multinomial logistic regression to analyze the correlates of metabolic diseases. And *p* < 0.05 was considered a statistically significant difference.

## Results

### The trajectories of metabolic diseases among nurses

**Figure 2:**
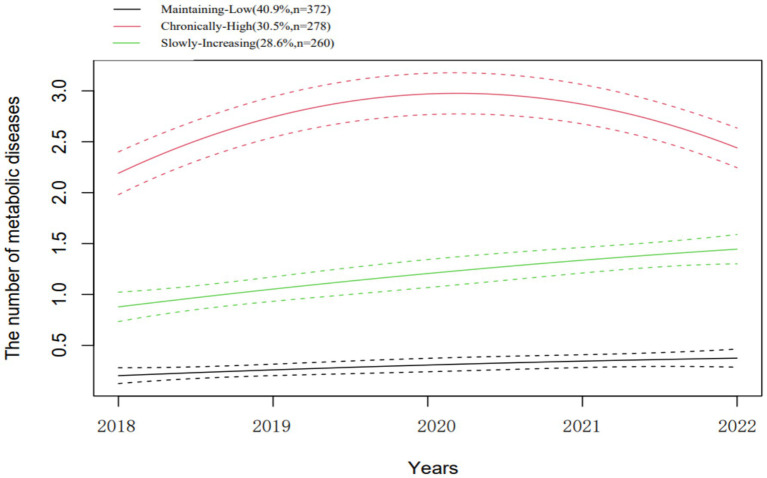
The trajectories of metabolic diseases.

We used the GMM to identify distinct groups of individuals sharing a similar trajectory of metabolic diseases. We tested from a one-class model to a five-class model. On the basis of the model-fitting indices and the interpretability of the classified trajectories, the three-class model showed the best fit (see Table 2), labeled as “Maintaining-Low,” “Chronically-High,” and “Slowly-Increasing.” Maintaining-Low group accounted for the highest proportion among nurses (40.9%, n = 372), and maintained a low number of metabolic diseases in 5 years. Nurses in Chronically-High group maintained high levels of metabolic diseases, although the number of metabolic diseases fluctuates during the follow-up period. Finally, the number of metabolic diseases among nurses in Slowly-Increasing group increased slowly with time (Figure 2).

**Table 2:** Fitted indices for GMMs with 1–5 classes.

Number of latent class	Model fit indices				Class category	
	LL	AIC	BIC	Entropy	*n* (%)	Posterior probability
1-class	−6199.55	12413.10	12446.79	/	910 (100)	1.00
2-class	−5984.49	11992.97	12050.73	0.6882	539 (59.2)	0.901
					371 (40.8)	0.934
3-class	−5924.17	11882.34	11964.17	0.6991	372 (40.9)	0.894
					278 (30.6)	0.935
					260 (28.6)	0.755
4-class	−5687.224	11818.45	11924.34	0.7184	364 (40.0)	0.910
					253 (27.8)	0.744
					248 (27.25)	0.889
					45 (4.95)	0.764
5-class	−5875.013	11804.03	11933.99	0.7518	364 (40.0)	0.921
					245 (26.9)	0.748
					41 (4.5)	0.775
					50 (5.5)	0.806
					210 (23.1)	0.840

### Characteristics associated with trajectories of metabolic diseases

The characteristics of the participants in each trajectory group for metabolic diseases are showed in Table 3. There were significant differences in gender, age, education level, marital status, years of service, smoking, dietary preference for vegetables, length of sleep and night shift pattern across trajectories (*p* < 0.05). Nurses in the “Chronically-High” trajectory group were more likely to be older, be male, be married and have more years of service and lower levels of education (*p* < 0.001 for all).

**Table 3:** Distribution of characteristics by metabolic diseases trajectories groups.

Variables		Maintaining-Low (*n* = 372)	Chronically-High group (*n* = 278)	Maintaining-Low group (*n* = 260)	*p* value
Gender	Female	367 (98.7)	242 (87.4)	252 (96.9)	<0.001
	Male	5 (1.3)	36 (12.6)	8 (3.1)	
Age		30.49 ± 5.77	35.79 ± 7.81	33.30 ± 6.95	<0.001
Education level	Technical secondary school and below	99 (26.6)	117 (42.1)	92 (35.4)	<0.001
	College and above	273 (73.4)	161 (57.9)	168 (64.6)	
Marital status	Not married	115 (30.9)	45 (16.2)	49 (18.8)	<0.001
	Married	257 (69.1)	233 (83.8)	2,111 (81.2)	
Years of service		13.77 ± 6.50	19.67 ± 9.12	17.01 ± 7.80	<0.001
Exercise	Yes	129 (34.7)	81 (29.1)	85 (32.7)	0.326
	No	243 (65.3)	197 (70.9)	175 (67.3)	
Drinking	Yes	31 (8.3)	29 (10.4)	23 (8.8)	0.645
	No	341 (91.7)	249 (89.6)	237 (91.2)	
Smoking	Yes	6 (1.6)	16 (5.9)	12 (4.6)	0.014
	No	365 (98.4)	256 (94.1)	248 (95.4)	
Regular diet	Yes	171 (46.0)	113 (40.6)	120 (46.2)	0.320
	No	201 (54.0)	165 (59.4)	140 (53.8)	
Dietary preference for vegetables	Yes	163 (43.8)	86 (30.9)	107 (41.2)	0.003
	No	209 (56.2)	192 (69.1)	153 (58.8)	
Length of sleep	7-8 h	185 (49.7)	98 (35.3)	121 (46.5)	0.001
	Other	187 (50.3)	180 (64.7)	139 (53.5)	
Night shift pattern	8-h night shift	205 (55.1)	169 (60.8)	162 (62.3)	0.027
	12-h night shift	116 (31.2)	71 (25.5)	52 (20.0)	
	Extended night shift	51 (13.7)	38 (13.7)	46 (17.7)	
Anxiety		12.91 ± 4.30	13.11 ± 4.50	13.38 ± 5.17	0.454
Depression		17.63 ± 6.52	17.78 ± 6.99	17.83 ± 7.02	0.927

### The relationship between healthy lifestyle, night shift pattern, mental health and metabolic diseases trajectories

The multinomial logistic regression was used to identify the relationship between lifestyle, night shift, mental health and metabolic diseases trajectories, and the results were showed in Table 4. After controlling covariates, the variables associated with the Chronically-High group included dietary preference for vegetables (OR = 0. 0.449, 95%CI: 0.306–0.658, *p* < 0.001) and exercise (OR = 0.515, 95%CI: 0.344–0.772, *p* < 0.05). The correlates of ‘Slowly-Increasing’ group were depression (OR = 0.928, 95%CI: 0.868–0.991, *p* < 0.05), anxiety (OR = 1.116, 95%CI: 1.015–1.228, *p* < 0.05), and 12-h night shifts (OR = 0.489, 95%CI: 0.287–0.834, *p* < 0.05).

**Table 4:** Multivariate logistic regression analysis with metabolic diseases’ trajectory.

Variables	Chronically-high			Slowly-increasing		
	(ref: maintaining-low)					
	OR	95% CI	*p*- value	OR	95% CI	*p*- value
Night shift pattern (ref: extended night shift)						
8-h night shifts	0.767	0.416–1.414	0.396	0.647	0.376–1.114	0.116
12-h night shifts	0.764	0.424–1.376	0.370	0.489	0.287–0.834	0.009
Health lifestyle						
Dietary preference for vegetables (ref: no)	0.449	0.306–0.658	<0.001	0.760	0.538–1.072	0.118
Regular diet (ref: no)	0.698	0.475–1.025	0.067	0.863	0.605–1.231	0.415
Length of sleep (ref: 7-8 h)	1.286	0.889–1.859	0.182	0.958	0.683–1.342	0.801
Smoking (ref: no)	1.187	0.367–3.841	0.775	1.966	0.655–5.904	0.228
Drinking (ref: no)	0.520	0.259–1.046	0.067	0.826	0.570–1.179	0.547
Exercise (ref: no)	0.515	0.344–0.772	0.001	0.820	0.567–1.172	0.284
Mental health						
Depression	0.961	0.898–1.027	0.241	0.928	0.868–0.991	0.027
Anxiety	1.043	0.945–1.151	0.402	1.116	1.015–1.228	0.024
Covariates						
Age	1.116	1.014–1.229	0.025	1.018	0.931–1.113	0.697
Marital status (ref: married)	0.614	0.387–0.976	0.039	0.645	0.427–0.976	0.038
Gender (ref: female)	34.098	10.640–109.327	<0.001	4.135	1.128–15.161	0.032
Education level (ref: college and above)	0.684	0.422–1.110	0.125	0.735	0.467–1.158	0.185
Years of service	1.059	0.969–1.157	0.207	1.059	0.975–1.151	0.175

## Discussion

As far as we know, it is the first study to pay attention to the trajectories of metabolic diseases among nurses. Using nurse cohort longitudinal data, we identified three distinct long-term trajectories of metabolic diseases development among nurse. Meanwhile, we associated lifestyle, night shift pattern and mental health with these metabolic diseases’ trajectories.

Some studies have confirmed the development of metabolic diseases among nurses, but we have not found the study of trajectories of metabolic diseases with time. Our findings confirmed the existence of heterogeneous metabolic diseases trajectories in Chinese nurse. And the three trajectories were ‘Maintaining-Low’, ‘Slowly- Increasing’ and ‘Chronically-High’. Similar results have been reported in other studies of multimorbidity trajectories (5, 13). Nurses in our study are generally young, but 59.1% of them were not in the’ Maintaining-Low’ group, and the health status of nurses is worrying. Previous studies have reported that nurses have a high level of metabolic diseases such as overweight/obesity and nonalcoholic fatty liver disease (14, 15), and they are on the rise (16). The existing study on nurses’ occupational health pays more attention to skeletal muscle diseases (17), however, the threat of nurses’ metabolic diseases to health needs attention and prevention. In addition, we did not find the trajectory of the gradual decline of the number of metabolic diseases. Most metabolic diseases (e.g., diabetes, hypertension), are often difficult to reverse completely to normal. Moreover, with a relatively short follow-up period (5 years), changes in disease counts may not be substantial enough to form distinct declining trajectories.

Among covariates, age, marital status and gender were correlates of different trajectory groups. Older nurses were more likely to be in the Chronically-High group. With aging, an increased prevalence of a clustering of metabolic abnormalities has been observed (18). The mechanism may be that the plasma level and the ability to produce proinflammatory cytokines generally increase with age (19), and the resulting chronic inflammation may be the key to the occurrence and development of metabolic diseases (20). Consistent with our findings, previous studies showed that the risk of metabolic disorder in males is greater than that in females in youth (18, 21). Notably, the exceedingly high odds ratio observed in this study is likely attributable to the small sample size of male participants. This limitation may have led to an overestimation of the effect size, and thus the finding should be regarded as preliminary until validated in larger, more adequately powered studies. Marriage may affect health. Unmarried men or women pay more attention to physical attractiveness to attract a partner, and women may be more affected by this (22). Besides, obesity is easily influenced by their spouse. If one spouse became obese, the likelihood that the other spouse would become obese increased by 37% (23).

In this study, nurses with exercise and dietary preferences for vegetables were more likely to belong to the Maintaining-Low group and have better health status. Lifestyle plays an important role in metabolic diseases, and lifestyle modification is an important part of the treatment of metabolic diseases, including regular exercise, healthy diet and so on (24). Different types of exercise have different effects on metabolism. In terms of body mass index, aerobic exercise is better than resistance exercise, and resistance exercise is the most effective in improving body fat, LDL-C level and SBP (25). Combined exercise is the best exercise program to improve body weight, DBP, TG, TC, glucose and insulin levels (25). In addition, the vegetable-based diet may lead to less energy intake and saturated fatty acids (26), thus helping to prevent and reduce the severity of metabolic diseases.

Interestingly, exercise and dietary preference for vegetables are not associated with the slow increase in metabolic disease count among nurses with high initial health level. And our results showed that mental health and night shift were found to be correlated with the trajectory of metabolic disease accumulation. We observed anxiety score was positively correlated with the increase in metabolic disease count, while depression score was negatively correlated. One possible explanation is that anxiety may activate the HPA axis, leading to cortisol dysregulation, insulin resistance, and visceral fat accumulation, thereby increasing metabolic risk (27). On the other hand, the burden of chronic metabolic diseases may also induce or exacerbate anxiety (28). The inverse association between depression and metabolic disease risk may reflect healthy worker/volunteer bias, as participants were likely healthier than the general population. Alternatively, nurses with depression score may seek healthcare more frequently, leading to earlier detection and management of metabolic abnormalities. 12-h night shifts were associated with a higher likelihood of belonging to the Maintaining-Low group. This suggests that the health effects of night shift work may depend more on the overall shift pattern than on the duration of a single shift. Compared to 8-h night shifts, 12-h night shifts typically involve fewer night shifts per month and a lower overall frequency. And 12-h shift workers were more frequently happy with their jobs, more likely to be satisfied with their shift schedule, less emotionally exhausted, and less likely to report missing shifts (29). In contrast to extended-night shifts, 12-h night shifts often incorporate a 4-h nap period. Previous study indicated that such on-duty napping positively impacts both hypertension and fatigue levels (30), aligning with evidence-based guidelines that recommend napping during night shifts as a fatigue countermeasure (31). Managers need to make efforts on nurses’ mental health and night shift work in order to maintain nurses’ health. Managers should pay attention to psychological screening and night shift scheduling patterns, and 12-h night shifts with a nap opportunity may be a preferable option.

### Limitations

There are several limitations of the current study that warrant discussion, and the results of this study should be interpreted with some caution. Firstly, our data is a retrospective cohort, and excluding 349 non-respondents and 290 participants with missing key data may have introduced selection bias. Nurses in poorer health may have been less likely to participate. And nurses with unhealthy lifestyles and poor mental health status may be reluctant to disclose private information, potentially resulting in missing data. Thus, the results may be more generalizable to nurses with higher participation and complete data. Secondly, lifestyle factors and mental health were assessed only at the end of the follow-up (2022). Although lifestyle habits in adults are generally considered stable, we cannot exclude the possibility of changes during the 2018–2022 period. Meanwhile, we were unable to establish the temporal direction of the relationship between psychological distress and the accumulation of metabolic diseases, and reverse causality cannot be ruled out. Third, using disease count as the outcome measure ignores the impact of disease severity on health. This approach dichotomizes continuous metabolic traits, making individuals near diagnostic thresholds susceptible to misclassification. Additionally, inconsistencies in diagnostic criteria across studies may limit the comparability of our findings. Future studies should adopt more comprehensive measures of disease burden, such as weighted multimorbidity indices or continuous metabolic risk scores, while also incorporating disease severity grading to evaluate metabolic health.

## 5 Conclusion

The changes of the number of metabolic diseases among nurses in China are heterogeneous, and the characteristics of trajectory group include’ Maintaining-Low’,’ Slowly-Increasing’ and’ Chronically-High’. Lack of dietary preference for vegetables and exercise are significantly related to nurses’ metabolic disorders. Among nurses with high initial health level, significant correlates of the increase in the number of metabolic diseases are not unhealthy lifestyles, but mental health and night shifts. The findings of this study suggest that managers should pay attention to the metabolic disorders of nurses, develop and provide interventions to manage and prevent metabolic diseases.

## Data Availability

The raw data supporting the conclusions of this article will be made available by the authors, without undue reservation.
