# Correction: The relationship between lifestyle, night shift pattern, mental health and the trajectories of nurses' metabolic diseases: a cohort study of nurses

**DOI:** 10.3389/fpubh.2026.1858252

**Published:** 2026-05-11

**Authors:** Min Yang, Ying Che, Jiaqi Xu, Xueqian Ma, Hongbo Chen, Baohua Li

**Affiliations:** 1 Department of Obstetrics and Gynecology, Peking University Third Hospital, Beijing, China; 2 Health Examination Center of Peking University Third Hospital, Beijing, China; 3 Nursing Department of Peking University Third Hospital, Beijing, China

**Keywords:** growth mixture model, metabolic diseases, nurse, population health, trajectories

Authors Min Yang and Ying Che were erroneously omitted as equal contributing authors.

The original version of this article has been updated.

